# Repeated biannual cross-sectional surveys in primary schools set baseline seasonal and spatial surveillance for malaria and schistosomiasis in the Shire Valley Transformation Programme (SVTP), Malawi

**DOI:** 10.1016/j.crpvbd.2026.100350

**Published:** 2026-01-06

**Authors:** Priscilla Kapolo, Blessings Chiepa, Rex B. Mbewe, Blessings Kapumba, Eggrey Kambewa, Lucy Kaunga, Sylvester Coleman, James Chirombo, Themba Mzilahowa, Christopher M. Jones, Michelle C. Stanton, J. Russell Stothard

**Affiliations:** a Malawi Liverpool Wellcome Research Programme, Blantyre, Malawi; b Liverpool School of Tropical Medicine, Liverpool, L3 5QA, UK; c Malaria Alert Centre, Kamuzu University of Health Sciences, Blantyre, Malawi; d Vestergaard S.A., Lausanne, Switzerland

**Keywords:** Surveillance, Public health, Malaria, Urogenital schistosomiasis, Intestinal schistosomiasis, Co-infection, Lower Shire Valley, Seasonality

## Abstract

Control of malaria and schistosomiasis among school children poses a key public health challenge in Chikwawa District, Malawi. Furthermore, anticipated environmental changes from the Shire Valley Transformation Programme (SVTP), a large-scale irrigation scheme, are expected to both alter transmission of malaria and schistosomiasis. To later inform future disease surveillance and appropriate control interventions, our study sought to establish comprehensive seasonal and spatial baseline epidemiological data. Four cross-sectional surveys were undertaken in 21 primary schools, covering two wet and two dry seasons. A total of 4176 children aged 7–13 years were examined using rapid diagnostic tests for malaria, urine reagent strips with egg-filtration microscopy for urogenital schistosomiasis, and urine-Circulating Cathodic Antigen (CCA) dipsticks for intestinal schistosomiasis. The overall prevalence was 10.8% (95% CI: 9.8–11.7%) for malaria, 36.5% (95% CI: 35.1–38.0%) for urogenital schistosomiasis, and 1.9% (95% CI: 1.5–2.4%) for intestinal schistosomiasis. Co-infection prevalence of malaria and urogenital schistosomiasis was 5.2% (95% CI: 4.5–5.9%). Macrohematuria was at 5.5% (95% CI: 4.8–6.2%) while microhematuria was at 26.2% (95% CI: 24.9–27.6%). Seasonal fluctuations were noted for malaria, whereas schistosomiasis was limited, although both diseases exhibited strong spatial heterogeneity. Alarmingly, malaria exceeded 25% and urogenital schistosomiasis surpassed 50% in certain schools, thus clearly demonstrating currently unmet public health needs. These are set to become further exacerbated by forthcoming SVTP-driven environmental change; hence, we provide critical evidence to guide the Malawi Ministry of Health in strengthening surveillance and preparing integrated disease control.

## Highlights

• Surveys of 4176 children revealed high malaria (10.8%) and schistosomiasis (36.5%) prevalence with 5.2% co-infection.
• Malaria varied seasonally, while schistosomiasis was seasonally stable, but both showed strong spatial heterogeneity.
• Some schools recorded malaria > 25% and urogenital schistosomiasis > 50%, indicating high local burden and hotspots.
• The findings provide critical evidence for integrated control ahead of Shire Valley (SVTP-driven) environmental changes.

## Graphical abstract

**Figure:**
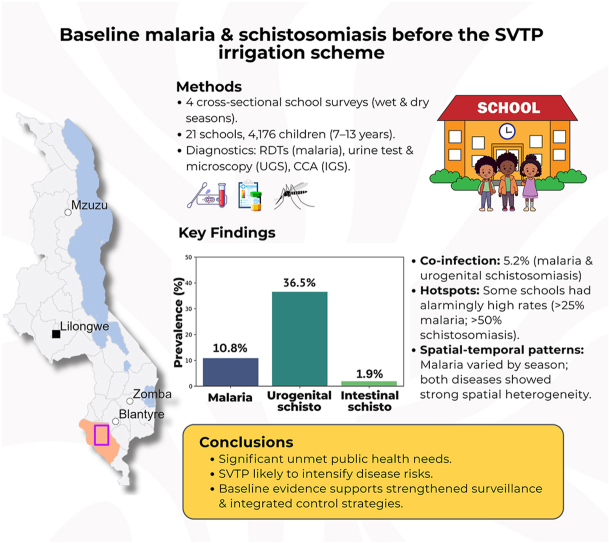

## 1 Introduction

Malaria and schistosomiasis are major vector-borne diseases with significant public health importance in sub-Saharan Africa (SSA), caused by protozoans and helminths, respectively (WHO, 2022). Malawi is among the 15 SSA countries with the highest malaria burden, reporting over four million cases in 2023 with no decline from 2015. In 2023, the estimated incidence increased by 3.1%, partly due to Cyclone Freddy, which expanded mosquito breeding habitats (WHO, 2024). Malaria prevalence is highest in humid, low-lying regions, particularly along Lake Malawi and the Lower Shire Valley, creating ideal conditions for *Anopheles* vectors (Chirombo et al., 2020). The National Malaria Control Programme (NMCP) conducts nationwide distribution of long-lasting insecticidal nets (LLINs), intermittent preventive treatment in pregnancy (IPTp), and recently rolled out the RTS, S/AS01 malaria vaccine for children under five (WHO, 2024). Through the National Malaria Strategic Plan (NMSP) 2023–2030, Malawi aims to eliminate malaria by 2030 in line with global targets (WHO, 2021). Emerging evidence also highlights the role of school-aged children as reservoirs of malaria transmission, indicating potential benefits of school-based chemoprevention strategies (Sixpence et al., 2024).

Schistosomiasis, a neglected tropical disease (NTD), remains highly prevalent among school-aged children in Malawi and contributes substantially to morbidity (Ministry of Health, 2024). Two main forms exist: urogenital schistosomiasis (caused by *Schistosoma haematobium*) and intestinal schistosomiasis (caused by *Schistosoma mansoni*), transmitted by aquatic snails of the genera *Bulinus* and *Biomphalaria*, respectively (Makaula et al., 2014). All districts in Malawi are endemic for schistosomiasis, with *S. haematobium* being most widely distributed, particularly along lakeshores and Lower Shire Valley River margins (Ministry of Health, 2024). Despite lower rates of intestinal schistosomiasis, recent reports indicate expansion of *Biomphalaria pfeifferi* into southern districts such as Chikwawa, posing an emerging health concern (Nkolokosa et al., 2025). Control relies primarily on mass drug administration (MDA) with praziquantel, following WHO preventive chemotherapy guidelines. In Chikwawa, MDA has been implemented biannually over the last decade, reflecting sustained endemicity and ongoing risk (Ministry of Health, 2024).

**Fig. 1:**
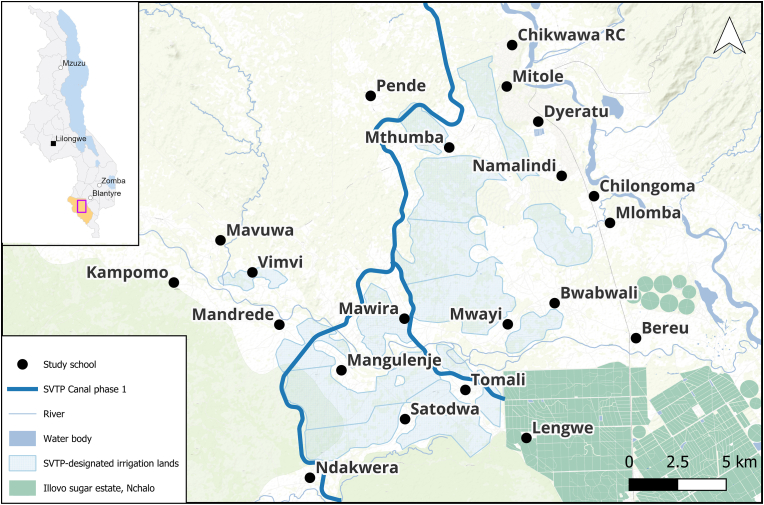
Map of the study area. Inset map shows the location of Chikwawa District within Malawi. On the main map, the thick blue line is the proposed irrigation canal, black dots represent the 21 primary schools, and the blocks of blue stripes are the designated irrigation blocks under SVTP Phase 1.

Chikwawa District, located in the Lower Shire Valley (Fig. 1), has environmental and socio-economic characteristics that make it particularly vulnerable to malaria and schistosomiasis transmission. The district lies at low elevation (30–150 m), experiences distinct wet and dry seasons with average temperatures around 30 °C and 740 mm annual rainfall, and has a population of 628,282 (Kasambala and Chinwada, 2011; NSO, 2024; Chikuse et al., 2025). The Shire River and its associated irrigation infrastructure, including large-scale sugarcane irrigation managed by Illovo Sugar, provide perennial water sources conducive to vector and snail proliferation.

Importantly, the government of Malawi, with support from the World Bank and partners, is implementing the Shire Valley Transformation Programme (SVTP), a 14-year initiative (2018–2031) that will construct 133 km of irrigation canals and transform over 40,000 ha for agriculture in Chikwawa and Nsanje (Gowernment of Malawi, 2023). Although irrigation activities have not yet begun, such large-scale land transformation is expected to alter vector ecology and potentially influence malaria and schistosomiasis transmission.

To establish a baseline for understanding the potential impact of SVTP on vector-borne diseases, the Shire Valley Vector Control Project (Shire-Vec) has been collecting data on vector populations and disease risk since September 2023. We now report findings from four bi-annual cross-sectional malaria and schistosomiasis parasitological surveys in primary schools in Chikwawa, aimed at comprehensively documenting seasonal fluctuations and spatial heterogeneity. The first survey in September 2023 revealed an alarming local disease burden (Chiepa et al., 2024), and we present results from three subsequent surveys conducted in March 2024, September 2024, and March 2025. The primary objective of this paper is to establish comprehensive seasonal and spatial baseline data on malaria and schistosomiasis among school-aged children in Chikwawa District, Malawi, to inform future surveillance and control strategies in the context of the SVTP.

## 2 Materials and methods

### 2.1 Study area

The cross-sectional study was conducted in 21 government-owned primary schools located within 3 km of the proposed SVTP Phase 1 irrigation canal in Chikwawa District. Four surveys were conducted between 2023 and 2025, repeated bi-annually in the same schools, purposefully aligned with the wet season from October to April and the dry season from May to September (DCCMS, 2025).

### 2.2 Recruitment of study participants

There are 22 government-owned and 2 private schools located within 3 km of the SVTP canal. Schools were included in the study if they had a population of at least 300 children aged 7–13 years. A total of 21 government-owned schools met this criterion and were enrolled in the study. Across the four cross-sectional surveys, a total of 4176 school-going children were enrolled in the study. Participants were randomly selected from school registers, and each was given information leaflets for their parents and guardians to read before the survey. Approximately 25 girls and 25 boys were selected, with a total target enrolment of 50 participants per school in each survey. The estimated population for the study area was 57,000, of which 15.2% were children in the age range 7–13 years (Government of Malawi, 2019). If a sample of 1050 (50 per school) was enrolled in the study, which represents 12.1% of the age group, it would provide an estimate of overall prevalence precision of 2.8% at a 95% confidence interval (CIESIN, 2020; Chiepa et al., 2024). As the design of the surveys was cross-sectional, there was no repeated enrolment of participants across all four surveys. In adherence with Good Clinical Practices, the study objectives and procedures were explained to the parents and guardians on the day of the survey before any study-related procedure was carried out to ensure informed parental/guardian consent was provided; after receipt of written informed consent, each child was enrolled in the study.

### 2.3 Study procedures

Participants in the study were tested on site for malaria, urogenital schistosomiasis, and intestinal schistosomiasis. Malaria testing was conducted using rapid diagnostic tests (RDT) from finger-prick blood using the Humasis Malaria Pf/PAN Antigen Test (Launch Diagnostics, Kent, UK). The manufacturer reports a relative sensitivity of > 99.9% and a relative specificity of > 99.5% for the Histidine-Rich Protein II (HRP-II) antigen, and > 95% and > 99.5%, respectively, for the *P. falciparum* lactate dehydrogenase (pLDH) antigen (Humasis, 2011). For schistosomiasis, the participants provided mid-morning, mid-stream urine that filled a 50-ml bottle and tested for intestinal schistosomiasis using the urine-Circulating Cathodic Antigen (CCA) lateral flow dipstick test (Rapid Medical Diagnostics, Pretoria, South Africa). According to the manufacturer, the sensitivity of the schistosomiasis POC-CCA test varies with infection intensity, ranging from 100% in high-intensity infections to approximately 70% in low-intensity infections (Schisto POC-CCA, 2018). While specificity was not reported by the manufacturer, Casacuberta-Partal et al. (2021) observed specificity of 95–99% in non-endemic populations. Visual hematuria (macrohematuria) was noted, and microhematuria was tested using a reagent strip (Multistix, Siemens, Manchester, UK). The CCA lateral flow test results were reported following the manufacturer’s recommendations on visual assessment of the colour intensity of the test line on the dipstick. Trace results were interpreted as positive on-site and confirmed in the laboratory using microscopy. All urine samples were then transported on the same day to the local laboratory in Chikwawa, where a 10-ml urine sample was filtered across a 25 μm nylon pore-sized circular nylon filter (1.3 cm in diameter). The nylon filters were inspected by microscopy (×100 magnification) according to WHO protocols with enumeration of eggs classified as absent, less than 10, 11–49, and more than 50.

### 2.4 Data collection

A simple structured questionnaire was also administered, capturing the demographics, insecticide-treated net (ITN) usage, water contact, health-seeking behaviours, malaria and schistosomiasis testing and treatment history for the past two years. Malaria and schistosomiasis test results were recorded using both paper-based forms (laboratory testing lists) and electronic forms in Open Data Kit (ODK), an electronic mobile data collection tool. For urogenital schistosomiasis, egg count data were recorded in ODK after urine microscopy. The collected data were uploaded to a secure ODK central server hosted by the Malawi Liverpool Wellcome (MLW) Research Programme in Blantyre for subsequent analyses.

### 2.5 Data analysis

Data were cleaned and analysed using R Statistical Software (Version 4.3) (R Core Team, 2024). The maps were created using QGIS Version 3.14. In addition to descriptive statistics, multivariate logistic regression models were fitted to evaluate the associations between the dependent variable (infection) and independent variables (age, ITN usage, water contact, treatment, and gender). The overall prevalence was calculated using a pooled analysis of data from all surveys, and spatial autocorrelation was calculated using Moran’s I test (Ogunsakin et al., 2024) for both infections. Confidence intervals (95% CI) were computed using the exact binomial Clopper-Pearson approach.

## 3 Results

### 3.1 Demographics and characteristics of study participants

A total of 4176 school-aged children were enrolled across the four surveys conducted between September 2023 and March 2025, with individual survey sample sizes ranging from 984 to 1134. The gender distribution was balanced, comprising 50.3% boys (*n* = 2090) and 49.7% girls (*n* = 2086). Participants were predominantly aged 8–12 years, with 12-year-olds representing the largest age group (30.4%), followed by those aged 10 (21.2%), 11 (20.5%), 9 (15.6%), and 8 (11.8%). Only a small proportion were aged 7 (0.1%) or 13 (0.3%). Full demographic characteristics are presented in Table 1.

**Table 1:** Demographics and characteristics of all study participants across four surveys. The total number and percentage of participants per survey and the overall total across four surveys are presented.

Variable	Response	September 2023	March 2024	September 2024	March 2025	Total
		*N* (%)	*N* (%)	*N* (%)	*N* (%)	*N* (%)
Gender	Male	554 (48.9)	504 (51.2)	510 (50.2)	518 (49.7)	2086 (49.9)
	Female	580 (51.1)	480 (48.8	505 (49.8)	525 (50.3)	2090 (50.1)
	Total	1134	984	1015	1043	4176
Age (years)	7	0 (0)	0 (0)	1 (0.1)	3 (0.3)	4 (0.1)
	8	141 (12.4)	113 (11.5)	114 (11.2)	125 (12.0)	493 (11.8)
	9	159 (14.0)	138 (14.0)	182 (17.9)	174 (16.7)	653 (15.6)
	10	231 (20.4)	199 (20.2)	246 (24.2)	210 (20.1)	886 (21.2)
	11	248 (21.9)	198 (20.1)	191 (18.8)	220 (21.1)	857 (20.5)
	12	355 (31.3)	336 (34.2)	278 (27.4)	301 (28.9)	1270 (30.4)
	13	0 (0)	0 (0)	3 (0.3)	10 (0.9)	13 (0.3)
Self-health assessment	Fine	1003 (88.4)	850 (86.4)	890 (87.7)	951 (91.2)	3694 (88.5)
	Not fine	131 (11.6)	134 (13.6)	125 (12.3)	92 (8.8)	482 (11.5)
ITN ownership	Yes	923 (81.4)	762 (77.4)	715 (70.4)	980 (94.0)	3380 (80.9)
	No	211 (18.6)	222 (22.6)	300 (29.6)	63 (6.0)	796 (19.1)
Slept in ITN last night	Yes	685 (60.4)	650 (66.1)	491 (48.4)	838 (80.3)	2664 (63.8)
	No	124 (10.9)	48 (4.9)	124 (12.2)	70 (6.7)	366 (8.8)
	No response	114 (10.1)	64 (6.4)	100 (9.8)	72 (7.0)	350 (8.3)
Tested for malaria previously	Yes	1114 (98.2)	861 (87.5)	800 (78.8)	921 (88.3)	3696 (88.5)
	No	20 (1.8)	123 (12.5)	215 (21.2)	122 (11.7)	480 (11.5)
Treated for malaria previously	Yes	1077 (95.0)	703 (71.4)	725 (71.4)	873 (83.7)	3378 (80.9)
	No	57 (5.0)	281 (28.6)	290 (28.6)	170 (16.3)	798 (19.1)
Play in water at home	Yes	603 (53.2)	444 (45.1)	459 (45.2)	568 (54.5)	2074 (49.7)
	No	531 (46.8)	540 (54.9)	556 (54.8)	475 (45.5)	2102 (50.3)
Play in water at school	Yes	45 (4.0)	42 (4.3)	3 (0.3)	28 (2.7)	118 (2.8)
	No	1089 (96.0)	942 (95.7)	1012 (99.7)	1015 (97.3)	4058 (97.2)
Treated for schistosomiasis previously	Yes	837 (73.8)	508 (51.6)	306 (30.1)	300 (28.8)	1951 (46.7)
	No	297 (26.2)	476 (48.4)	709 (69.9)	743 (71.2)	2225 (53.3)
Last PZQ treatment	This school year	528 (46.6)	18 (1.8)	34 (3.3)	17 (1.6)	597 (14.3)
	Last school year	156 (13.8)	448 (45.5)	237 (23.3)	218 (20.9)	1059 (25.4)
	Previous years	153 (13.5)	42 (4.3)	35 (3.4)	65 (6.2)	295 (7.1)
	No treatment	297 (26.2)	476 (48.4)	709 (69.9)	743 (71.2)	2225 (53.3)

Most participants (88.5%; 3694/4176) reported feeling well on the day of data collection. Water-contact behaviour was common, with 50.3% (2102/4176) reporting contact with water sources either at home, at school, or in both locations. Most of the exposure occurred at home. Reported waterbodies included rivers, dams, canals, rainwater pools, and localised stagnant sources such as *thamanda*, *thawale*, and *zithaphwi*. These terms describe water found within agricultural ridges (*thamanda* and *thawale*) and stationary muddy water or swamp-like areas (*zithaphwi*). Participants also identified several commonly accessed perennial and seasonal waterbodies, including the rivers Shire, Mthumba, and Khuku, as well as irrigation canals and dams. Additional frequencies for all variables are available in Table 1.

### 3.2 Temporal and seasonal trends in malaria and schistosomiasis prevalence

**Fig. 2:**
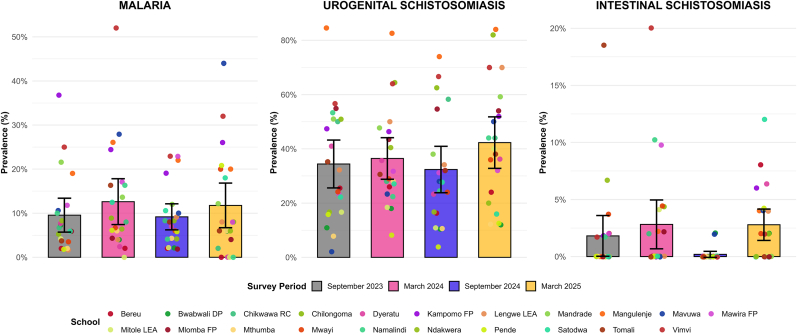
Prevalence of malaria, urogenital and intestinal schistosomiasis infections across the four surveys conducted between September 2023 and March 2025. Solid bars represent the overall prevalence with 95% confidence intervals (lines). Coloured dots uniquely identify each school participating in the survey.

Malaria prevalence showed seasonal variation over the two-year surveillance period (Fig. 2). The overall malaria prevalence across the four surveys was 10.8% (449/4176, 95% CI: 9.8–11.7%). A relatively higher burden was recorded in surveys conducted during the wet season, with prevalence peaking at 12.5% in the March 2024 survey (123/984, 95% CI: 10.5–14.7%) and at 11.8% in the March 2025 survey (123/1043, 95% CI: 9.9–13.9%). In contrast, lower infection rates were reported during the dry season, with a prevalence of 9.7% (110/1134, 95% CI: 8.0–11.6%) and 9.2% (93/1015, 95% CI: 7.5–11.1%) in September 2023 and 2024, respectively. Out of the 21 primary schools, 17 reported malaria cases across all surveys, with the highest prevalence of 52% (26/50) at Vimvi (March 2024), 44% (22/50) at Mavuwa (March 2025) and 36.8% (21/57) at Kampomo (September 2023).

**Fig. 3:**
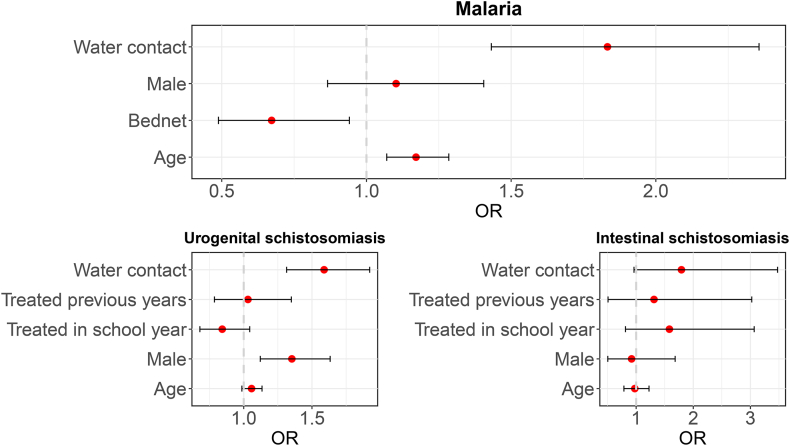
Forest plots showing adjusted odds ratios (OR) and 95% confidence intervals from the multivariable logistic regression model for malaria infection and urogenital and intestinal schistosomiasis. The vertical dotted lines represent the null value (OR = 1).

Interestingly, Mitole LEA primary school, located in the urban setting closer to Chikwawa district hospital, showed an unusual infection pattern; malaria cases were reported in the dry season only: in September 2023 (4.3%, 2/47, 95% CI: 0.5–14.5%) and 2024 (2.2%, 1/46, 95% CI: 0.1–11.5%). Each additional year of age was associated with a 15% significant increase in the odds of malaria infection (OR = 1.15, 95% CI: 1.07–1.24, *P* < 0.001), and as shown in Fig. 3, boys had 26% higher odds of infection compared to girls (OR = 1.26, 95% CI: 1.03–1.54, *P* = 0.023). The children who played with water had 82% higher odds of becoming infected (OR = 1.82, 95% CI: 1.49–2.24, *P* < 0.001).

There was a moderate endemicity for urogenital schistosomiasis with an overall prevalence of 36.5% (1525/4176, 95% CI: 35.1–38.0%). The prevalence of the baseline survey (September 2023) was 35.0% (397/1134, 95% CI: 32.2–37.9%), which drastically increased to 42.4% (442/1043, 95% CI: 39.4–45.4%) in the March 2025 survey. These epidemiological surveys showed alarming high number of cases that were reported across all schools with the highest number of cases reported in the following schools located closer to the proposed SVTP canal: Mangulenje (81.4%, 166/204, 95% CI: 75.3–86.5%), and Vimvi (63.9%, 133/208, 95% CI: 57.0–70.5%) and in another school located near Shire River and the main road namely, Chilongoma (64.5%, 129/200, 95% CI: 57.4–71.1%). The lowest prevalences across all surveys were seen at Pende school (10.2%, 21/206, 95% CI: 6.4–15.2%), located to the north side of the proposed SVTP canal and designated irrigation lands and Bwabwali school (12.8%, 26/203, 95% CI: 8.5–18.2%) located near the SVTP designated irrigation lands. Male participants had 30% higher odds of getting urogenital schistosomiasis infection compared to female participants (OR: 1.30, 95% CI: 1.15–1.48, *P* < 0.001). There was a significant association between age and urogenital schistosomiasis infection; with each additional year of age, the odds of infection increased by 8% (OR = 1.08, 95% CI: 1.03–1.13, *P* < 0.001) as shown in Fig. 3. The prevalence of microhematuria was 26.2% (95% CI: 24.9–27.6%), higher than the prevalence of macrohematuria of 5.5% (95% CI: 4.8–6.2%). Children who had water contact had 66% higher odds of infection (OR: 1.66, 95% CI: 1.46–1.88, *P* < 0.001).

Intestinal schistosomiasis was less prevalent than urogenital schistosomiasis with an overall prevalence rate of 1.9% (80/4176, 95% CI: 1.5–2.4%) over the 2-year period (Fig. 2). The prevalence during September 2023 surveys was 1.9% (21/1134, 95% CI: 1.1–2.8%) which slightly increased to 2.9% (28/984, 95% CI: 1.9–4.1%) in the March 2024 survey. Despite the overall lower prevalence, Vimvi (5.8%, 12/208, 95% CI: 3.0–9.9%) and Tomali (4.9%, 10/203, 95% CI: 2.4–8.9%), located around Lengwe National Park, showed higher prevalences. The lowest prevalence (only 1 positive case) was reported in schools located furthest from the SVTP, Chikwawa RC, Mlomba and Mthumba. One school, located near Shire River, Lengwe National Park and the designated irrigation lands, Mwayi Primary School, consistently reported intestinal schistosomiasis cases across 3 surveys (2.5%, 5/199, 95% CI: 0.8–5.8%). The odds of intestinal schistosomiasis infection among children were not significantly associated with either gender (OR = 1.13, 95% CI: 0.73–1.77, *P* = 0.586) or age (OR = 1.12, 95% CI: 0.95–1.33, *P* = 0.196). Children who had water contact showed 27% higher odds of infection (OR: 1.27, 95% CI: 0.81–1.99, *P* = 0.303) (Fig. 3).

### 3.3 Dynamics of malaria and schistosomiasis co-infection

Malaria and urogenital schistosomiasis co-infection was also assessed in this study, with an overall prevalence of 5.2% (215/4176, 95% CI: 4.5–5.9%). The prevalence of co-infection in the September 2023 survey was 5.5% (62/1134, 95% CI: 4.2–7.0%), and it dropped to 3.5% (36/1015, 95% CI: 2.5–4.9%) in the September 2024 survey before rising again to 5.8% (61/1043, 95% CI: 4.5–7.4%) in the March 2025 survey. Higher co-infection rates were seen in schools that were endemic to both malaria and urogenital schistosomiasis, located along the proposed SVTP canal and Mwanza River namely, Vimvi (21.6%, 45/208, 95% CI: 16.2–27.9%), Mangulenje (16.2%, 33/204, 95% CI: 11.4–22.0%), and Kampomo (12.3%, 24/195, 95% CI: 8.0–17.8%). A relatively lower prevalence (0.5%) was recorded at Bereu and Mitole LEA Primary School.

### 3.4 Spatial distribution and patterns of malaria and schistosomiasis

**Fig. 4:**
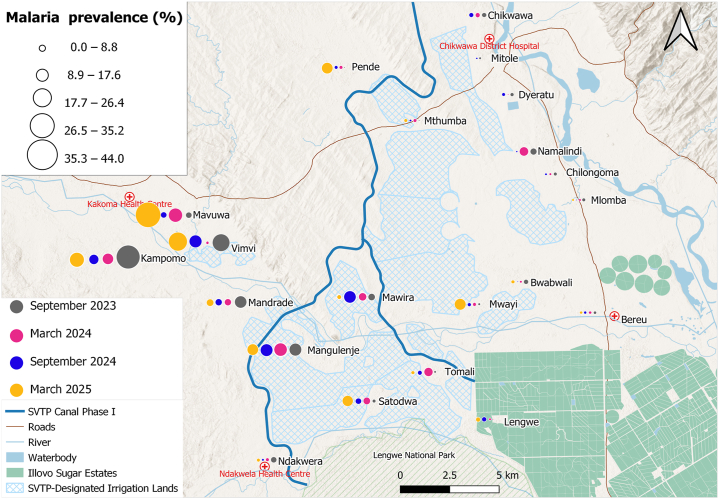
Spatial trends of malaria prevalence across the four surveys. The circles represent the overall prevalence of infection at a school per survey.

**Fig. 5:**
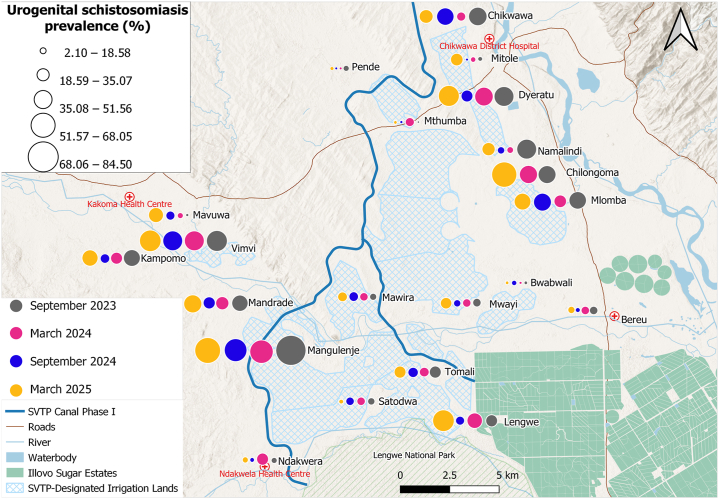
Spatial trends of egg-patent urogenital schistosomiasis prevalence across the four surveys. The circles represent the overall prevalence of infection at a school per survey.

**Fig. 6:**
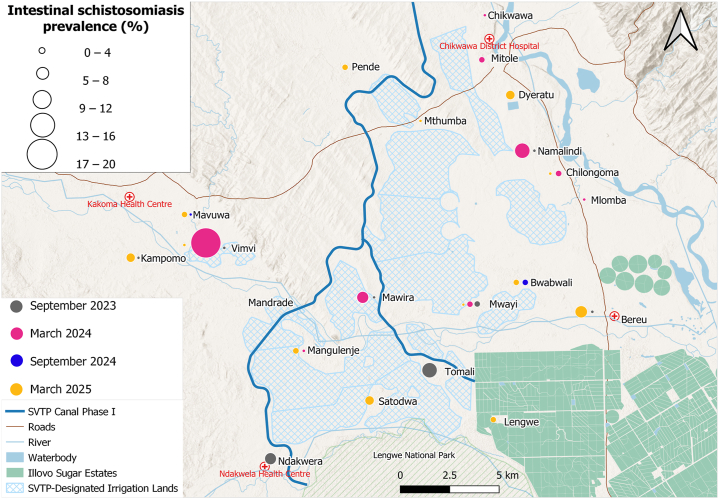
Spatial trends of intestinal schistosomiasis prevalence across the four surveys. The circles represent the overall prevalence of infection at a school per survey.

**Fig. 7:**
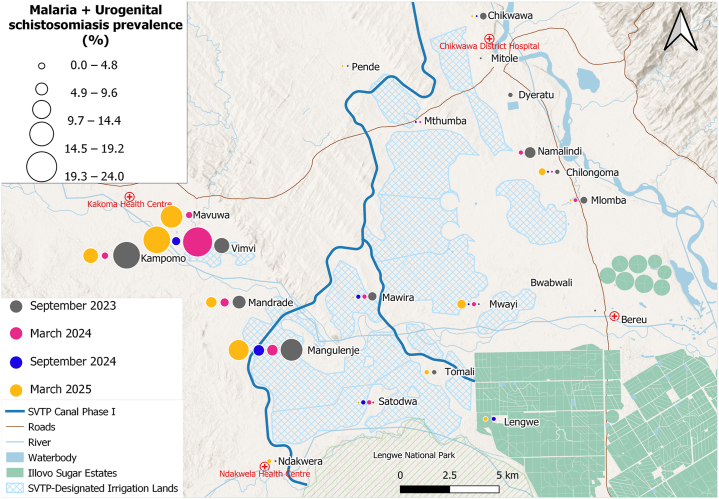
Spatial trends of malaria and urogenital schistosomiasis co-infection prevalence across the four surveys. The circles represent the overall prevalence of infection at a school per survey.

Malaria infection (Fig. 4) was elevated in schools located west of the proposed SVTP irrigation canal and Mwanza River, within the Kakoma Health Centre catchment area, with prevalences of 32.7% (68/208) at Vimvi*,* 27.2% (53/195) at Kampomo, 23.2% (44/190) at Mavuwa, 14.9% (29/194) at Mandrade, and 21.6% (44/204) at Mangulenje. Malaria prevalence exhibited a strong positive spatial clustering among schools (Moran’s I = 0.674, *P* < 0.001), indicating that schools with high prevalence were geographically proximate to one another. In contrast, egg-patent urogenital schistosomiasis (Fig. 5) was widespread across all 21 schools, with the highest prevalence reported in schools along the proposed SVTP canal, particularly at Mangulenje (81.4%, 166/204) and Vimvi (63.9%, 133/208) and those located near the Shire River: Chilongoma (64.5%, 129/200) and Mlomba (52%, 104/200). Lower infection rates were observed in schools near Lengwe National Park and the Illovo Sugar Estates. No significant spatial autocorrelation was observed at the school-level (Moran’s I = 0.018, *P* = 0.297). Intestinal schistosomiasis prevalence (Fig. 6) was low across most schools, though infection cases were reported at Vimvi (5.8%), Tomali (4.9%) and Satodwa (3%), all located around the Illovo Estate’s irrigation canals and dams. There was no significant spatial autocorrelation of infection across the 21 schools (Moran’s I = – 0.007, *P* = 0.363). The spatial distribution of malaria and schistosomiasis co-infection (Fig. 7) showed a higher burden concentrated in schools located to the west of the proposed SVTP canal and around irrigation-designated lands. There was a significant positive spatial autocorrelation in co-infection rates among schools (Moran’s I = 0.451, *P* < 0.001), suggesting that schools with high co-infection rates tend to be located near each other.

Water-contact behaviour remains a major challenge for schistosomiasis control. Half of all participants (50.3%; 2102/4176) reported water contact at home, school, or both, with most exposure occurring at home. Children reported interacting with a variety of water bodies, including rivers (67.3%), dams (11.5%), canals (7.2%), rainwater pools (1.2%), and small stagnant sources such as *thamanda*, *thawale*, and *zithaphwi*. Frequently mentioned perennial water sources included the Shire, Mthumba, and Khuku rivers, as well as irrigation canals and dams associated with agricultural schemes.

## 4 Discussion

Our study established comprehensive seasonal and spatial baseline data on malaria and schistosomiasis among 4176 school-aged children in Chikwawa District. Malaria prevalence was 10.8%, urogenital schistosomiasis 36.5%, and intestinal schistosomiasis 1.9%, with 5.2% co-infection of malaria and urogenital schistosomiasis. Malaria showed some seasonal variation and widespread distribution across schools, with notable spatial clustering west of the proposed SVTP canal. In contrast, schistosomiasis exhibited limited seasonal change but substantial spatial heterogeneity, exceeding 50% prevalence in some schools. These patterns appear linked to local ecological features, including nearby rivers and dams that are likely sustaining vector and intermediate host habitats. Collectively, our findings highlight significant unmet public health needs that may intensify with forthcoming SVTP-driven environmental changes.

Fewer infected cases were observed in the schools furthest from the proposed SVTP canal and situated in the urban setting of the district. For example, the malaria cases observed at Mitole primary school were from surveys conducted in the dry seasons, only showing unusual patterns that need further investigation. These lower prevalences are likely associated with socio-economic factors that have an impact on the prevalence of malaria infection (Larson et al., 2021). As expected, malaria infection showed seasonality patterns with more infections recorded in surveys conducted in wet seasons (March) than in dry seasons (September). These findings align with studies from Malawi that report higher malaria incidence during the rainy season (e.g. Hajison et al., 2017; Gondwe et al., 2021; Wafula et al., 2024).

Furthermore, the NMCP has been conducting ITN campaigns since 2012, distributing ITNs across districts in Malawi and this was recently done in 2025 in Chikwawa. ITNs remain the primary malaria prevention strategy in Malawi, supported by periodic mass distribution campaigns; first in 2012, expanded nationally in 2016, and most recently in 2023 and January 2025. Across all four surveys, household ITN ownership was high at 80.9% (3380/4176), well above the national average of 55% (NMCP, 2022). Ownership exceeded 70% in all survey rounds and peaked at 93.9% (980/1043) in March 2025. Among participants who owned an ITN, 78.8% (2664/3380) reported sleeping under one the night before the survey, although 10.8% did not and 10.4% were unsure. Despite this high ownership and reported use, 9.3% (247/2664) of ITN users still tested positive for malaria, and malaria positivity among ITN owners in March 2025 (10.8%) did not decline proportionately with the increase in coverage. This suggests that factors beyond ITN coverage, such as seasonal transmission intensity and inconsistent ITN use, may have influenced infection patterns. Ownership of an ITN was associated with 33% lower odds of malaria infection (OR = 0.67, 95% CI: 0.543–0.854, *P* < 0.001), and sleeping under an ITN reduced the odds of infection by 33% compared with non-use (OR = 0.673, 95% CI: 0.4956–0.9487, *P* = 0.017).

These findings highlight important gaps in protection and indicate that ITNs alone may be insufficient to provide optimal malaria prevention for school-aged children in these settings. As observed in other studies, self-reported ITN use may overestimate actual adherence, with objective measurements showing over-reporting by approximately 13.6% (Krezanoski et al., 2018). Additionally, the vector species mediating transmission may increasingly evade ITN protection by shifting biting behaviour. *Anopheles* spp. mosquitoes in similar settings have been documented to bite outdoors (Killeen, 2014) or into the morning hours when children are already in school. In western Kenya, peak landing rates of *Anopheles funestus* in schools occurred between 6:00 and 7:00 h and continued until 11:00 h, coinciding with school hours; more than half of the collected mosquitoes were fed or gravid, suggesting multiple blood meals per gonotrophic cycle and contributing to sustained transmission (Omondi et al., 2023). Such behavioural and ecological dynamics may partly explain the persistence of infection despite high ITN coverage in the present study.

Malaria prevalence was comparatively lower among participants attending schools near Lengwe National Park and the Illovo Sugar Estate Plantations. This could largely be due to the complementary measure implemented by Illovo Sugar of indoor residual spraying (IRS), suggesting that this integrated approach can effectively reduce the risk of infection among school-aged children, as also reported by Kambiring’oma et al. (2025). Other control interventions have also been introduced, such as school-based malaria case management, where children are diagnosed and tested using RDTs and treated with ACT by trained teachers in primary schools, as observed in a trial study in Zomba (Mphwatiwa et al., 2017). This initiative can increase access to early diagnosis and treatment of infections in school-aged children. A regression model developed in this study revealed that the risk of malaria infection increases with age; similarly, Coalson et al. (2016) had also reached the same conclusions from their study. These observations also support the notion of a shift in the malaria infection reservoir from under-fives to the school-aged children as reported by Mathanga et al. (2015). This shift highlights the need for the Ministry of Health and NMCP to include school-aged children in the integrated and targeted vector control strategies (Walldorf et al., 2015; Spaans et al., 2024; Mbewe et al., 2023).

Mass drug administration (MDA) with praziquantel remains the primary schistosomiasis control strategy in Chikwawa, as elsewhere in Malawi, with the most recent district-wide campaign conducted in 2023. In our surveys, 46.7% (1951/4176) of children reported receiving praziquantel during the current or previous school year, leaving more than half untreated. Among those treated, 14.3% received treatment in the current school year, 25.4% in the previous school year, and 7.1% in earlier years. Despite this, infection prevalence remained high: 35.4% (691/1951) of treated children were still positive, including 200 treated in the same school year and 491 treated in prior years. These findings align with the consistently moderate-to-high prevalence of urogenital schistosomiasis across all schools, underscoring the persistent endemicity of the infection. As expected for a chronic condition, urogenital schistosomiasis exhibited limited seasonality, likely sustained by continuous exposure to perennial waterbodies such as the Shire River, Illovo Estate irrigation canals, and local dams, where malacological surveys confirmed the presence of intermediate host snails (Nkolokosa et al., 2025). Persistent infections may reflect rapid reinfection due to ongoing water contact, incomplete or unreliable self-reported treatment histories, suboptimal treatment coverage, variation in drug uptake, or reduced drug efficacy in field settings. Collectively, these results highlight that while MDA remains essential, it is insufficient on its own to interrupt transmission without complementary interventions aimed at reducing water exposure, strengthening health education, and addressing the environmental drivers that sustain transmission.

The persistence of high-risk water-contact behaviour among school-aged children remains a challenge to schistosomiasis control in Chikwawa District. Our findings indicate that half of all participants reported exposure, predominantly at home, with rivers constituting the most frequented waterbodies. This environmental exposure is compounded by significant behavioural and knowledge gaps; consistent with other studies, a general lack of community understanding regarding schistosomiasis prevention and life cycle underscores the critical need for robust health education integrated with vector control initiatives (Koffi et al., 2018; Rassi et al., 2019; Lubanga et al., 2024). The resulting disease burden appears relatively higher than anticipated by the current national control strategy, which classifies Chikwawa as a moderate-prevalence area warranting mass drug administration (MDA) only once every two years. However, to align with the WHO (2022) guidelines and progress towards the 2030 elimination goal, we recommend an escalation of MDA frequency to at least an annual basis. This requires dedicated resource mobilisation for the National Control Programme. Furthermore, a singular reliance on MDA is insufficient. A comprehensive, multi-pronged strategy is essential, combining increased chemotherapy with targeted environmental and behavioural interventions. This includes focal vector control in perennial water sources like the irrigation canals and Shire River, community-led water, sanitation, and hygiene (WASH) programmes, and targeted test-and-treat campaigns in high-prevalence school hotspots identified in this study.

The observed age-related infection pattern, where older children showed a higher risk of urogenital schistosomiasis, a finding consistent with Reed et al. (2023), further suggests that control programmes must extend their focus beyond early primary school years to include older adolescents. Even though the prevalence of intestinal schistosomiasis was low in this study, the emergence of the *Biomphalaria pfeifferi* snails in Chikwawa poses a future public health concern (Nkolokosa et al., 2025). This is already observed at Tomali primary schools, one of the study schools that had a higher prevalence of intestinal schistosomiasis compared to other schools and where these snails have already been detected. Amidst a moderate prevalence of egg-patent urogenital schistosomiasis, this might lead to an endemicity of intestinal schistosomiasis among the school-aged children as reported in Mangochi District (Kayuni et al., 2020). There is a need for a proactive approach to surveillance and control of the snail vectors since there are numerous freshwater waterbodies and holding reservoirs/dams in Chikwawa, which are conducive for colonisation by this snail if given the opportunity. While chemotherapy is effective for both forms of schistosomiasis, environmental control must be tailored to the differing snail vectors, as a single strategy is unlikely to be effective for both. The highest prevalence of intestinal schistosomiasis was observed at Vimvi, a school that revealed higher prevalence for all infections that were tested in this study. Both infections can be targeted and treated concurrently.

Co-infection of malaria and egg-patent schistosomiasis was more elevated in schools located on the west side of the district, as compared to the other schools, where the co-infection prevalence was lower. Control efforts should be integrated to address both infections simultaneously, particularly as the same schools, such as Vimvi and Mangulenje, were found to carry a high burden of both diseases. Unlike other studies that did not test for both infections, this study covers such gaps and limitations. However, there might be a possible relationship between the two infections in terms of environmental drivers for vector distribution and spread which this study did not assess and must be investigated.

This study found that both malaria and schistosomiasis exhibited spatial heterogeneity across schools, although only malaria showed significant clustering in specific locations. In contrast, urogenital schistosomiasis prevalence was not significantly clustered at the school level, likely reflecting widespread and ubiquitous water contact among children rather than the distribution of intermediate snail hosts. These findings highlight the need to complement bi-annual MDA with targeted interventions, including health education, water contact reduction strategies, and environmental measures such as snail control. Malaria clustering was particularly pronounced in schools near the proposed SVTP canal, suggesting that the development of the canal could increase mosquito breeding habitats and elevate transmission risk.

This study also has several limitations that should be acknowledged. First, its cross-sectional design precludes causal inference and limits the ability to determine temporal relationships between environmental factors and infection prevalence. Secondly, reliance on self-reported information, such as insecticide-treated net (ITN) use and praziquantel treatment history, may have introduced recall or social desirability bias, potentially affecting the accuracy of these variables. Finally, as the surveys were school-based, the findings may not fully represent infection dynamics among non-enrolled or younger children and the wider community, particularly adults who may also contribute to ongoing transmission. Notwithstanding these limitations, the study provides valuable and comprehensive baseline data to inform future longitudinal and community-wide surveillance as the Shire Valley Transformation Programme progresses.

Despite these limitations, the findings highlight clear priorities for public health action. Malawi’s Ministry of Health should focus on targeted interventions at the district level, prioritising highly burdened schools and surrounding villages where transmission remains intense. Such data-driven interventions and policies can help reduce infection intensity and interrupt transmission in the most affected areas. To support the 2030 malaria elimination and schistosomiasis control and elimination goals, there is a need to strengthen and complement existing strategies, as recommended by the WHO (2022). These may include integrated vector management (IVM), larval source management (LSM), and indoor residual spraying (IRS) for malaria (Gowelo et al., 2020), alongside measures to reduce contact with freshwater bodies, snail control, and environmental management for schistosomiasis (Makaula et al., 2025). Furthermore, SVTP stakeholders, the Ministry of Health, and development partners should invest in integrated control measures to reduce vector distribution and the spread of infection among school-aged children living near the proposed irrigation canals in Chikwawa District.

## 5 Conclusions

There is a clear need to control vector-borne diseases such as malaria and schistosomiasis amidst extending agricultural productivity, especially through large-scale irrigation schemes to achieve both economical and health objectives. Despite ongoing control measures, chronic malaria detected by finger-prick RDTs remains a public health issue among school-aged children in Chikwawa and school-based malaria chemoprevention programmes will likely be needed in future as the SVTP completes. Egg-patent urogenital schistosomiasis remains alarmingly prevalent, posing an immediate detrimental health threat to the school-aged population, with the likely future emergence of intestinal schistosomiasis. Upscale for preventive chemotherapy is needed alongside initiatives that prevent re-exposure/infection. The Ministry of Health at the district level should focus on a more targeted approach for vector control, with particular attention on implementing snail control measures targeting *Biomphalaria* species and management by targeting highly burdened schools rather than generalised targeting of ITNs and praziquantel. SVTP should set financial resources for vector control and management as the irrigation canal opens for operation.

## CRediT authorship contributions statement

**Priscilla Kapolo:** Formal analysis, Writing – original draft, Investigation, Visualization. **Blessings Chiepa:** Conceptualization, Methodology, Formal analysis, Investigation, Writing – original draft, Writing - review & editing, Visualization. **Rex B. Mbewe:** Conceptualization, Methodology, Investigation, Writing – review & editing. **Blessings Kapumba:** Investigation. **Eggrey Kambewa:** Investigation. **Lucy Kaunga:** Investigation. **Syvester Coleman:** Investigation, Writing - review & editing. **James Chirombo:** Supervision, Writing – review & editing. **Themba Mzilahowa:** Writing – review & editing, Supervision, Funding acquisition. **Christopher M. Jones:** Writing – review & editing, Supervision, Funding acquisition. **Michelle C. Stanton:** Conceptualization, Writing – review & editing, Supervision, Funding acquisition. **J. Russell Stothard:** Conceptualization, Methodology, Investigation, Resources, Writing – review & editing, Supervision, Funding acquisition.

## Ethical approval

The study was approved by the College of Medicine’s Research Ethics Committee (Protocol number: P.03/23/4041) and the Liverpool School of Tropical Medicine Research Ethics Committee (Protocol number: 22–039). In adherence with Good Clinical Practices, the study objectives and procedures were explained to the parents and guardians on the day of the survey before any study-related procedure was carried out to ensure informed parental/guardian consent was provided. After receiving written informed consent, each child was enrolled in the study. Additional details can be found in the baseline survey paper by Chiepa et al. (2024).

## Funding

This study and article are funded by the NIHR (NIHR Global Health Research Group on Controlling Vector-Borne Diseases in Emerging Agricultural Systems in Malawi, grant number NIHR 133144). The views expressed are those of the author(s) and not necessarily those of the NIHR.

## Declaration of competing interests

The authors declare that they have no known competing financial interests or personal relationships that could have appeared to influence the work reported in this paper.

## Data Availability

The data supporting the conclusions of this article are included within the article. Raw data can be available upon a request to the corresponding author.
